# Safety and Tolerability of KIO-101 Eye Drops in Healthy Volunteers and Patients with Ocular Surface Disease—A Phase I Study

**DOI:** 10.3390/pharmaceutics16030367

**Published:** 2024-03-05

**Authors:** Doreen Schmidl, Nikolaus Hommer, Martin Kallab, Andreas Schlatter, Clemens Nadvornik, Franz Obermayr, Stefan Sperl, Eric J. Daniels, Gerhard Garhöfer

**Affiliations:** 1 Department of Clinical Pharmacology, Medical University of Vienna, 1090 Vienna, Austria; doreen.schmidl@meduniwien.ac.at (D.S.); nikolaus.hommer@oegk.at (N.H.); martin.kallab@kepleruniklinikum.at (M.K.); a.schlatter@viros.at (A.S.);; 2 VIROS—Vienna Institute for Research in Ocular Surgery—Karl Landsteiner Institute, Hanusch Hospital, 1140 Vienna, Austria; 3 Kiora Pharmaceuticals, Inc., Encinitas, CA 92024, USA; obermayr.franz@gmail.com (F.O.); ssperl@kiorapharma.com (S.S.); edaniels@kiorapharma.com (E.J.D.)

**Keywords:** ocular surface disease, KIO-101, DHODH inhibitor, safety, efficacy

## Abstract

Purpose: Inhibitors of dihydroorotate dehydrogenase (DHODH) have been found to be potent anti-inflammatory agents. Recently, a topical formulation (KIO-101 eye drops) of a DHODH inhibitor has been developed. The aim of the present study was to evaluate the safety and tolerability of KIO-101 eye drops in Healthy Volunteers (HVs) and patients with conjunctival hyperemia. Methods: The study was carried out in a double-masked, placebo-controlled, randomized, parallel-group design with two parts. In part I, HVs received single and multiple instillations (four times daily for 12 consecutive days) of KIO-101 eye drops in ascending doses of 0.05%, 0.15%, and 0.30%, respectively. Part II was conducted in patients with conjunctival hyperemia who received 0.15% KIO-101 eye drops twice daily for 12 consecutive days. Ophthalmic and systemic safety examinations were performed on all participants. In part II, ocular hyperemia grading and an ocular surface disease index (OSDI) questionnaire were performed. Results: 24 HVs participated in part I and 21 patients in part II. KIO-101 eye drops were well tolerated in all subjects. No serious adverse events (SAEs) occurred, and all AEs that were reported were transient and considered mild to moderate. In the highest dose cohort (0.30%), epistaxis occurred in two subjects after multiple instillations. In part II, after 12 days treatment with 0.15% KIO-101, conjunctival hyperemia decreased by −1.1 ± 0.27 points in the treatment and −0.6 ± 0.79 points in the placebo group (*p* = 0.0385). OSDI decreased from 47.9 ± 18.7 to 27.6 ± 19.13 points in the treatment group, while in the placebo group, a change from 41.3 ± 12.08 to 27.3 ± 18.63 points occurred. Conclusions: A 12-day treatment regimen with topical KIO-101 eye drops at low and mid doses was safe and well tolerated in both HVs and patients with conjunctival hyperemia. The obtained results point towards an early sign of reduction in conjunctival hyperemia.

## 1. Introduction

A variety of ocular conditions are associated with ocular surface inflammation, such as dry eye disease (DED) and infectious or allergic conjunctivitis [1,2]. In order to reduce inflammation and restore ocular surface integrity, several therapeutic agents are available including steroids, non-steroidal anti-inflammatory drugs, and others [3]. These approaches come with established side effects such as elevated intraocular pressure after chronic use, increased risk of cataract formation, or a limited safety profile in patients with a compromised corneal surface [3,4]. Thus, there is still a medical need for safe and well-tolerated broad-spectrum anti-inflammatory agents for the treatment of ocular inflammatory conditions. 

Inhibitors of dihydroorotate dehydrogenase (DHODH) have been suggested as a promising option. DHODH is a mitochondrial inner membrane enzyme involved in the de-novo synthesis of pyrimidine nucleotides [5]. Depending on the affected cells, inhibitors of DHODH therefore can exert a variety of different actions including immunosuppressive, antiproliferative, and also antiviral effects [5]. Leflunomide and its metabolite teriflunomide, which are oral inhibitors of DHODH, are approved for the treatment of rheumatoid arthritis and relapsing multiple sclerosis but have also been found to be beneficial in other autoimmune diseases such as myasthenia gravis or systemic lupus [5,6,7]. Recent studies also report a promising effect in the treatment of neoplasms as well as in viral infections such as COVID-19 [8,9,10,11].

KIO-101, formerly known as PP-001, is a recently developed, small-molecule inhibitor of DHODH with high potency [12]. Intravitreal application of KIO-101 in a rat experimental model of uveitis showed promising results, an effect that has mainly been attributed to reduced T-cell-dependent inflammation [12]. The aim of the present clinical study was to test the safety and tolerability of a topical formulation of the DHODH inhibitor KIO-101. In this first-in-human (FIH) study, KIO-101 eye drops were administered to healthy volunteers and patients with conjunctival hyperemia. Ocular safety and measures of conjunctival hyperemia were assessed after single and multiple dose administration. 

## 2. Materials and Methods

### 2.1. Study Population

The study protocol was approved by the Ethics Committee of the Medical University of Vienna, the national competent authorities, and was conducted at the Department of Clinical Pharmacology in compliance with the Declaration of Helsinki and Good Clinical Practice (GCP) guidelines of the European Union. All healthy volunteers and patients provided written informed consent before any study-related procedures were performed. 

In the two weeks before the first study day, all study participants were required to pass screening examinations consisting of the following procedures: ophthalmic examination including visual acuity testing, slit lamp examination, dilated funduscopy, visual field and Amsler grid testing, physical examination, blood draw for routine laboratory assessment, vital signs, and electrocardiogram (ECG). In patients for part II of the study, ocular surface disease index (OSDI) questionnaire was performed in addition to the general slit lamp examination. All women of childbearing potential were required to undergo a pregnancy test. In order to be included, all study participants had to be generally healthy, with no clinically significant abnormal findings in the physical examination, laboratory testing, and ECG. In order to participate in part I of the study, subjects also had to have no abnormal observations at the ophthalmic examination including dry eye. To be included in part II, patients had to present an OSDI score of 22 or more and conjunctival hyperemia of at least grade 2 on the Efron Scale in both eyes [13].

### 2.2. Study Design

**Figure 1:**
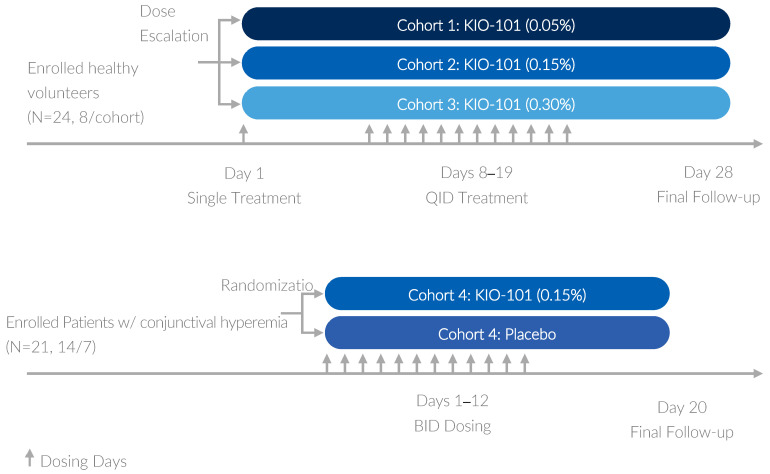
Time schedule.

The study was carried out in a double-masked, placebo-controlled, randomized, parallel-group design in two different parts. In part I, safety and tolerability of KIO-101 in ascending dosages was investigated in 3 cohorts of 8 healthy volunteers (HVs) randomized to receive KIO-101 or placebo (6:2, respectively). In part II, safety and tolerability were tested in a single cohort of 21 patients with conjunctival hyperemia who were randomized to receive KIO-101 or vehicle (2:1, respectively). An overview of the study design is shown in Figure 1.

- Part I

Dose escalation was performed in three individual dose cohorts consisting of 8 HVs in each cohort. Eligible HVs in the first cohort were randomized to receive a single drop of 0.05% KIO-101 eye drops or vehicle in one eye. After a break of at least one week after single-dose administration, the identical cohort of HVs received KIO-101 or vehicle at the same dose 4 times daily for 12 consecutive days. Safety and tolerability assessments were performed throughout the study. The same study schedule was followed for cohort 2 (KIO-101 at 0.15% or placebo) and cohort 3 (KIO-101 at 0.30% or placebo), respectively. In addition to safety assessments, blood samples for pharmacokinetic (PK) analysis of KIO-101 in plasma were collected at the following time points: Day 0 pre-dose, 30 min and 60 min after instillation, Day 8 and Day 19, each 30 min after the last instillation of the day.

- Part II

Based on the results of part I, the second-highest dose of 0.15% KIO-101 was chosen for part II of the study. In part II, 21 patients with conjunctival hyperemia were randomized to receive either KIO-101 eye drops or vehicle for 12 consecutive days twice daily with at least 8 h in between instillations into one randomized eye. Since only one assigned eye was treated, patients were provided with a bottle of a topical lubricant (Genteal HA, Laboratoires Thea, Clermont-Ferrand, France) for the non-study eye as needed. Patients were instructed to document instillation dates and times for Genteal HA eye drops in a patient diary.

### 2.3. Investigational Medical Product (IMP) and Placebo

KIO-101 eye drops consisted of the pharmacologically active ingredient (3-{[2,3,5,6-tetrafluoro-3′-(trifluoromethoxy)biphenyl-4-yl] carbamoyl}thiophene-2-carboxylic acid, previously known as PP-001) formulated in sodium chloride, sodium hydroxide, hydrochloric acid, 5% human serum albumin, and water for injection. As a control, the vehicle for KIO-101 eye drops was formulated as KIO-101 without the pharmacologically active substance.

### 2.4. Safety Assessments

To assess safety, the number, frequency, and severity of adverse events (AEs) were recorded throughout the study. Clinical laboratory testing (including hematology, chemistry, and coagulation status), physical examinations, vital signs (heart rate, systolic and diastolic blood pressure), and 12-lead ECGs were also performed at predefined time points.

To investigate ocular safety, best-corrected visual acuity testing was carried out using an Early Treatment Diabetic Retinopathy Study (EDTRS) chart; slit lamp examination and indirect fundoscopy were performed to examine the conjunctiva, cornea, and anterior chamber; and a 30° visual field measurement was conducted using automated perimetry (Humphrey Field Analyzer, Zeiss, Germany) with an Amsler grid assessment. Finally, intraocular pressure (IOP) was measured using Goldmann applanation tonometry with prior instillation of fluorescein and oxybuprocain.

- Additional assessments (Part II only)

To further investigate the safety and tolerability of KIO-101 in patients with conjunctival hyperemia, the Ocular Surface Disease Index (OSDI) was employed to interrogate the frequency of specific symptoms and their impact on vision-related functioning [14]. In addition, conjunctival hyperemia was assessed by the investigator during slit lamp examination according to the scale proposed by Efron et al. 1998 (0 = none, 1 = trace, 2 = mild, 3 = moderate, 4 = severe) [13].

### 2.5. Statistical Analysis

Descriptive analyses of continuous variables (summary statistics) are presented with the number of non-missing observations, arithmetic mean, and standard deviation (±SD). Categorical variables (frequency statistics) are presented with the number of non-missing observations and percentages (%). Percentages were calculated within each stratum on the total number of non-missing observations. Adverse Events were coded according to MedDRA. MedDRA-preferred terms and system organ class were used for AE summaries. For post hoc analyses of efficacy parameters for cohort 4 (KIO-101 eye drops vs. placebo), Wilcoxon tests for continuous variables were used. For changes in conjunctival hyperemia, an additional ANCOVA model was used with a treatment effect term and baseline value as a covariate.

A *p*-value < 0.05 was considered statistically significant.

## 3. Results

A total of 63 HVs and patients with conjunctival hyperemia were screened for eligibility, of which 45 were eligible and enrolled in the present study. In total, 24 HVs participated in Part I and 21 patients with conjunctival hyperemia in part II, respectively. Of the total number of 45 study participants, 49% were male and 51% female. The baseline characteristics of each of the study cohorts are provided in Table 1. No SAEs occurred during the course of the study in any HV or patient. A total of 83 AEs were reported, of which none were considered severe, while 75 were considered mild and 8 moderate. Of all AEs, none were judged as definitely related to KIO-101 eye drops; one (1) AE was probably related, 45 AEs were possibly related, and 37 were considered as not related. Based on the results of part I of the study, the second-highest dose of 0.15% KIO-101 was chosen for part II of the study. Detailed descriptions for part I and II of the study are provided below.

### 3.1. Part I (Healthy Volunteers)

KIO-101 eye drops and placebo were well tolerated on the ocular surface in all cohorts. No safety signs were observed at the slit lamp examination except mild conjunctival hyperemia at some single time points in single subjects (*n* = 5 for the treated eye, *n* = 2 for the placebo eye, and *n* = 3 for the untreated eye). No abnormalities were found at visual field testing, Amsler grid evaluation, or indirect fundoscopy at any visit. BCVA and IOP remained stable throughout the study. In one subject, slightly elevated IOP was observed at one time point in both, the treated and untreated eye, and unblinding after completion of the study revealed that this subject had received a placebo. In terms of systemic safety, no clinically significant changes in laboratory values, vital signs, or physical examination occurred in any of the subjects. However, in cohort 3, in two subjects, epistaxis occurred after multiple instillations. Although these AEs were considered non-severe and moderate, cohort 3 was terminated by the sponsor and no further subjects were included after the occurrence of these AEs.

In general, the most common AEs in cohorts 1–3 (*n* = 24) were headache (seven subjects with eight AEs), nasopharyngitis (five subjects with six AEs), rhinitis (four subjects with four AEs), and epistaxis (two subjects with three AEs). 

In the cohort 1–3 placebo group (*n* = 6), the most common AE was nasopharyngitis (two subjects with four AEs). A detailed listing of AEs that occurred in cohorts 1–3 is provided in Table 2.

#### Systemic Pharmacokinetics

A total of 125 plasma samples for analysis of PK parameters were obtained from participants in part 1 of the study, of which 90% (*n* = 113) were found to be below the lower limit of detection (LLOD < 10 ng/mL). In the 12 samples that were above the LLOD, the highest value observed was 48.0 ng/mL.

### 3.2. Part II (Patients with Conjunctival Hyperemia)

The 0.15% KIO-101 eye drops and vehicle were well tolerated in patients with conjunctival hyperemia. No safety signs of clinical concern were observed at slit lamp examination except for mild lid edema and erythema at some time points, but these were present already at the screening visit and tended to decrease during the course of the study (*n* = 7 at baseline and *n* = 3 after-12 days treatment in treated eyes, and *n* = 4 at baseline and *n* = 0 in vehicle eyes after 12 days treatment). Similarly, after 12 days treatment with 0.015% KIO-101, OSDI decreased from 47.9 ± 18.7 to 27.6 ± 19.13 points in the treatment group, while in the vehicle group, a change from 41.3 ± 12.08 to 27.3 ± 18.63 points occurred. Differences between groups were not significant.

**Figure 2:**
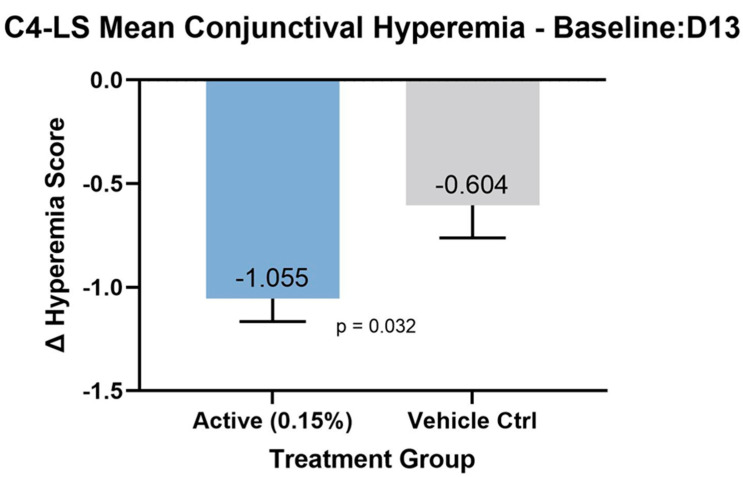
Mean reduction in conjunctival hyperemia score after 12 days treatment.

In total, 14 of 14 (100%) patients in the treatment group achieved at least a reduction of −1 or greater from baseline for conjunctival hyperemia, compared to 3 of 7 (43%) receiving control. This difference was found to be statistically significant between groups (*p* = 0.006). The absolute change from baseline to Day 13 for the conjunctival hyperemia assessment was −1.1 ± 0.27 in the treatment vs. −0.6 ± 0.79 in the placebo group (*p* = 0.0385, Figure 2).

The most common AEs in cohort 4 (total *n* = 21) were foreign body sensation in eyes (five subjects with eight AEs), and eye pruritus (two subjects with nine AEs). All AEs were transient and had resolved by the end of the study. Details are provided in Table 3.

## 4. Discussion

The results of this FIH study indicate that topical KIO-101 eye drops in the selected dosages are well tolerated and safe in both healthy subjects and patients with conjunctival hyperemia. Further, preliminary data in patients indicate a beneficial effect of KIO-101 eye drops by reducing conjunctival hyperemia.

PP-001, the pharmacologically active ingredient in KIO-101 eye drops, is a third-generation small-molecule inhibitor of dihydroorotate dehydrogenase (DHODH). Chemically speaking it has the molecular formula C_19_H_8_F_7_NO_4_S and a molecular weight of 479.3 g/mol. PP-001 is 150-fold more potent than other orally administered small-molecule DHOHD inhibitors, such as leflunomide, which currently holds regulatory approval for the treatment of rheumatoid arthritis and multiple sclerosis. As its basic mechanism of action, the inhibiting of DHODH leads to a pronounced suppression of the expression of inflammatory cytokines. As such, a previous study in a rat model showed that PP-001 strongly downregulates suppression of the cytokines IFN-γ, IL-17, TNF-α, IL-10, and IL-13, all of which have been found to play a role in several ocular inflammatory diseases including but not limited to ocular surface disease [12,15]. Independently, highly proliferating T cells are inhibited, which explains the strong anti-inflammatory effect of the drug. This is of particular interest given that T-cells have been found to play a central role in ocular inflammatory conditions and, therefore, a topically applied DHOHD inhibitor represents an attractive therapeutic [16,17].

In the current study, we report on the safety and local tolerability of KIO-101 eye drops in different concentrations after single and repeated instillation over 12 days in both HVs and patients with conjunctival hyperemia. Overall, data from this study indicate that KIO-101 eye drops were both safe and tolerable after single and repeated instillation over 12 days in both HVs and patients with conjunctival hyperemia. The most common finding at the slit lamp examination was conjunctival redness in healthy volunteers. These findings of redness were mild and transient and are frequent side effects of eye drops in general. Further, no clinically relevant change in visual field testing, Amsler grid evaluation, or indirect funduscopy occurred during the course of the study. IOP remained stable except for a single report of elevated IOP in both eyes (treated and untreated) in a single HV who received the placebo. 

With respect to systemic safety, no SAEs occurred throughout the study and the majority of AEs that were reported were considered as mild. No clinically relevant changes in laboratory values, vital signs, or physical examination were observed in any of the dose cohorts. In this context, it needs to be pointed out that systemic absorption of KIO-101 eye drops was very low, since concentrations of the active product ingredient were below the limit of quantification in the majority of plasma samples. In the highest dose cohort (0.30%), epistaxis was observed in two of six subjects receiving KIO-101 shortly after the instillation. This observation led to a discontinuation of the dosage group 0.3%. However, no changes in laboratory parameters were observed in these subjects and also blood pressure was within normal limits. As this event occurred only in two HVs, it is unclear whether it is directly related to KIO-101.

Safety and tolerability were also assessed in patients with conjunctival hyperemia. As for the healthy group, KIO-101 was well tolerated in this group of patients. Further, a significant reduction in conjunctival hyperemia in the study eye was found, when compared to the placebo. This points towards an anti-inflammatory effect of KIO-101 eye drops and warrants further investigation in adequately powered studies. 

In conclusion, 12-day treatment with topical KIO-101 eye drops at 0.05 and 0.15% is both safe and well tolerated in both healthy volunteers and patients with ocular surface inflammation.

## Figures and Tables

**Table 1:** Baseline characteristics of the study groups.

	Cohort 1 PP-001 0.05% Active (*n* = 6)	Cohort 2 PP-001 0.15% (*n* = 6)	Cohort 3 PP-001 0.30% (*n* = 6)	Cohort 1–3 Combined (*n* = 18)	Cohort 4 PP-001 0.15% (*n* = 14)	Cohort 1–3 Placebo (*n* = 6)	Cohort 4 Placebo (*n* = 7)	All Subjects (*n* = 45)
Height (cm)	177.2 ± 15.52	178.5 ± 6.69	174.7 ± 6.02	176.8 ± 9.87	172.1 ± 10.40	180.0 ± 5.62	167.3 ± 5.79	174.3 ± 9.69
Weight (kg)	76.3 ± 11.55	76.2 ± 14.55	76.2 ± 5.23	76.2 ± 10.47	79.2 ± 18.47	78.8 ± 5.31	78.3 ± 12.54	77.8 ± 13.02
Gender								
Male (*n* (%))	3 (50.0)	4 (66.7)	4 (66.7)	11 (61.1)	3 (21.4)	6 (100)	2 (28.6)	22 (48.9)
Female (*n* (%))	3 (50.0)	2 (33.3)	2 (33.3)	7 (38.9)	11 (78.6)	0 (0.0)	5 (71.4)	23 (51.1)
Childbearing potential								
Yes (*n* (%))	3 (100)	2 (100)	2 (100)	7 (100)	7 (63.6)	0 (NC)	2 (40.0)	16 (69.6)
Smoking habits								
non-smoker (*n* (%))	5 (83.3)	2 (33.3)	4 (66.7)	11 (61.1)	5 (35.7)	5 (83.3)	1 (14.3)	22 (48.9)
smoker (*n* (%))	1 (16.7)	4 (66.7)	1 (16.7)	6 (33.3)	6 (42.9)	0 (0.0)	5 (71.4)	17 (37.8)
ex-smoker (*n* (%))	0 (0.0)	0 (0.0)	1 (16.7)	1 (5.6)	3 (21.4)	1 (16.7)	1 (14.3)	6 (13.3)

Data are presented as mean ± SD. *n*…number of subjects, percentages are based on total. Percentages for childbearing potential are based on female participants.

**Table 2:** Listing of Adverse Events that occurred in groups 1–3.

	Cohort 1 Verum (*n* = 6)	Cohort 2 Verum (*n* = 6)	Cohort 3 Verum (*n* = 6)	Cohort 1–3 Verum (*n* = 18)	Cohort 1–3 Placebo (*n* = 6)
Infections and infestations	4 (66.7) 4	1 (16.7) 1	2 (33.3) 2	7 (38.9) 7	2 (33.3) 3
Nasopharyngitis	2 (33.3) 2	1 (16.7) 1	0 (0.0) 0	3 (16.7) 3	2 (33.3) 3
Rhinitis	2 (33.3) 2	0 (0.0) 0	2 (33.3) 2	4 (22.2) 4	0 (0.0) 0
Nervous system disorders	2 (33.3) 2	1 (16.7) 1	3 (50.0) 6	6 (33.3) 9	1 (16.7) 1
Headache	2 (33.3) 2	1 (16.7) 1	3 (50.0) 4	6 (33.3) 7	1 (16.7) 1
Dysgeusia	0 (0.0) 0	0 (0.0) 0	2 (33.3) 2	2 (11.1) 2	0 (0.0) 0
Eye disorders	0 (0.0) 0	0 (0.0) 0	0 (0.0) 0	0 (0.0) 0	1 (16.7) 4
Foreign body sensation in eyes	0 (0.0) 0	0 (0.0) 0	0 (0.0) 0	0 (0.0) 0	1 (16.7) 1
Eye pruritus	0 (0.0) 0	0 (0.0) 0	0 (0.0) 0	0 (0.0) 0	1 (16.7) 1
Eye discharge	0 (0.0) 0	0 (0.0) 0	0 (0.0) 0	0 (0.0) 0	1 (16.7) 1
Eye irritation	0 (0.0) 0	0 (0.0) 0	0 (0.0) 0	0 (0.0) 0	1 (16.7) 1
Gastrointestinal disorders	2 (33.3) 4	1 (16.7) 2	0 (0.0) 0	3 (16.7) 6	0 (0.0) 0
Abdominal pain	1 (16.7) 1	1 (16.7) 1	0 (0.0) 0	2 (11.1) 2	0 (0.0) 0
Diarrhea	1 (16.7) 1	1 (16.7) 1	0 (0.0) 0	2 (11.1) 2	0 (0.0) 0
Nausea	1 (16.7) 1	0 (0.0) 0	0 (0.0) 0	1 (5.6) 1	0 (0.0) 0
Vomiting	1 (16.7) 1	0 (0.0) 0	0 (0.0) 0	1 (5.6) 1	0 (0.0) 0
General disorder and administration site conditions	0 (0.0) 0	1 (16.7) 1	0 (0.0) 0	1 (5.6) 1	0 (0.0) 0
Fatigue	0 (0.0) 0	1 (16.7) 1	0 (0.0) 0	1 (5.6) 1	0 (0.0) 0
Injury, poisoning, and procedural complications	1 (16.7) 1	0 (0.0) 0	1 (16.7) 1	2 (11.1) 2	0 (0.0) 0
Heat stroke	0 (0.0) 0	0 (0.0) 0	1 (16.7) 1	1 (5.6) 1	0 (0.0) 0
Thermal burn	1 (16.7) 1	0 (0.0) 0	0 (0.0) 0	1 (5.6) 1	0 (0.0) 0
Respiratory, thoracic, and mediastinal disorders	1 (16.7) 1	0 (0.0) 0	2 (33.3) 3	3 (16.7) 4	0 (0.0) 0
Epistaxis	0 (0.0) 0	0 (0.0) 0	2 (33.3) 3	2 (11.1) 3	0 (0.0) 0
Oropharyngeal pain	1 (16.7) 1	0 (0.0) 0	0 (0.0) 0	1 (5.6) 1	0 (0.0) 0
Reproductive system and breast disorders	0 (0.0) 0	0 (0.0) 0	1 (16.7) 1	1 (5.6) 1	0 (0.0) 0
Dysmenorrhea	0 (0.0) 0	0 (0.0) 0	0 (0.0) 0	0 (0.0) 0	0 (0.0) 0
Genital discomfort	0 (0.0) 0	0 (0.0) 0	1 (16.7) 1	1 (5.6) 1	0 (0.0) 0

Values represent number of subjects (left side), percentages based on number of subjects (in brackets), total number of events (right side).

**Table 3:** Listing of Adverse Events that occurred in group 4.

	Cohort 4 Verum (*n* = 14)	Cohort 4 Placebo (*n* = 7)
Nervous system disorders	0 (0.0) 0	3 (42.9) 6
Headache	0 (0.0) 0	2 (28.6) 5
Tremor	0 (0.0) 0	1 (14.3) 1
Eye disorders	6 (42.9) 15	1 (14.3) 12
Foreign body sensation in eyes	4 (28.6) 6	1 (14.3) 2
Eye pruritus	1 (7.1) 1	1 (14.3) 8
Eye discharge	1 (7.1) 1	0 (0.0) 0
Asthenopia	0 (0.0) 0	1 (14.3) 1
Conjunctival hyperemia	1 (7.1) 1	0 (0.0) 0
Conjunctival irritation	1 (7.1) 2	0 (0.0) 0
Corneal erosion	1 (7.1) 1	0 (0.0) 0
Eye pain	0 (0.0) 0	1 (14.3) 1
Lacrimation increased	1 (7.1) 1	0 (0.0) 0
Vision blurred	1 (7.1) 1	0 (0.0) 0
Vitreous floaters	1 (7.1) 1	0 (0.0) 0
Gastrointestinal disorders	0 (0.0) 0	2 (28.6) 2
Abdominal pain	0 (0.0) 0	1 (14.3) 1
Diarrhea	0 (0.0) 0	0 (0.0) 0
Nausea	0 (0.0) 0	1 (14.3) 1
General disorders and administration site conditions	1 (7.1) 1	2 (28.6) 2
Influenza-like illness	0 (0.0) 0	1 (14.3) 1
Instillation site paresthesia	0 (0.0) 0	1 (14.3) 1
Pain	1 (7.1) 1	0 (0.0) 0
Injury, poisoning, and procedural complications	0 (0.0) 0	1 (14.3) 1
Arthropod sting	0 (0.0) 0	1 (14.3) 1
Reproductive system and breast disorders	1 (7.1) 1	0 (0.0) 0
Dysmenorrhea	1 (7.1) 1	0 (0.0) 0
Cardiac disorders	0 (0.0) 0	1 (14.3) 1
Tachyarrhythmia	0 (0.0) 0	1 (14.3) 1
Investigations	0 (0.0) 0	1 (14.3) 2
Intraocular pressure increased	0 (0.0) 0	1 (14.3) 2
Skin and subcutaneous tissue disorders	0 (0.0) 0	1 (14.3) 2
Pruritus	0 (0.0) 0	1 (14.3) 2

Values represent number of subjects (left side), percentages based on number of subjects (in brackets), total number of events (right side).

## Data Availability

Dataset available on request from the authors.
